# A Guide to the Practical Study of Diseases of the Eye; with an Outline of Their Medical and Operative Treatment

**Published:** 1860-04

**Authors:** 


### BOOK AND PAMPHLET NOTICES.

A GUIDE TO THE PRACTICAL STUDY OF DISEASES OF THE EYE: With an outline of their Medical and Operative Treatment. BY JAMES DIXON, F. R. C. S., Surgeon to the Royal London Opthalmic Hospital, etc.. etc. From the Second London Edition. Philadelphia: Lindsay & Blakiston. 1860.

This is the title to a small sized octavo volume of 427 pages. It is strictly an elementary treatise, or manual, and we think a very useful one. Its chapters are brief, but direct and practical; and their recommendations, so far at least as we have been able to examine them, judicious.

